# Zeolite-based Impedimetric Gas Sensor Device in Low-cost Technology for Hydrocarbon Gas Detection

**DOI:** 10.3390/s8127904

**Published:** 2008-12-05

**Authors:** Sebastian Reiß, Gunter Hagen, Ralf Moos

**Affiliations:** Bayreuth Engine Research Center, University of Bayreuth, 95440 Bayreuth, Germany E-Mails: Sebastian.Reiss@Uni-Bayreuth.de; Gunter.Hagen@Uni-Bayreuth.de; Ralf.Moos@Uni-Bayreuth.de

**Keywords:** OBD (On-Board-Diagnosis), electroplating, HC, VOC

## Abstract

Due to increasing environmental concerns the need for inexpensive selective gas sensors is increasing. This work deals with transferring a novel zeolite-based impedimetric hydrocarbon gas sensor principle, which has been originally manufactured in a costly combination of photolithography, thin-film processes, and thick-film processes to a low-cost technology comprising only thick-film processes and one electroplating step. The sensing effect is based on a thin chromium oxide layer between the interdigital electrodes and a Pt-loaded ZSM-5 zeolite film. When hydrocarbons are present in the sensor ambient, the electrical sensor impedance increases strongly and selectively. In the present work, the chromium oxide film is electroplated on Au screen-printed interdigital electrodes and then oxidized to Cr_2_O_3_. The electrode area is covered with the screen-printed zeolite. The sensor device is self-heated utilizing a planar platinum heater on the backside. The best sensor performance is obtained at a frequency of 3 Hz at around 350 °C. The good selectivity of the original sensor setup could be confirmed, but a strong cross-sensitivity to ammonia occurs, which might prohibit its original intention for use in automotive exhausts.

## 1. Introduction

In order to meet the steadily increasing emission regulations, which limit the tailpipe emission of carbon monoxide (CO), nitrogen oxides (NO_x_), and hydrocarbon (HC) [1], current gasoline-fueled automobiles are equipped with at least one three way catalyst (TWC). Since the TWC catalyst needs stoichiometric operation conditions, an oxygen sensor in the exhaust is required to provide an electrical signal indicating whether the engine is running rich or lean [2]. The need for a reliable “On-Board Diagnosis” (OBD) of the exhaust gas aftertreatment system enforces an additional sensor downstream catalyst to monitor its functionality [3]. Today's diagnosis strategies rely on the fact that the oxygen storage capacity is correlated with the conversion efficiency. Applying a second oxygen sensor downstream of the TWC allows to deduce indirectly the state and the efficiency of the catalytic converter [4]. However, in future cars with lowest emissions such an indirect method might not be accurate enough to decide whether the exhaust gas aftertreatment system meets the emission requirements [5]. Therefore, a direct measurement method employing a sensor that detects the amount of unburnt hydrocarbons is desirable. Such a hydrocarbon sensor can also be used for On-Board Diagnosis of close-coupled oxidation catalysts in diesel engines [6].

Recently, a zeolite-based thick-film sensor was suggested as a HC exhaust gas sensor [7-9]. Since zeolites are already in use for catalyst purposes in the exhaust, especially as a hydrocarbon adsorbing material to store cold-start hydrocarbon emissions until the TWC reaches its light-off temperature (e.g. [10-15]), zeolite-based sensors might be suitable to withstand the harsh conditions in the exhaust.

## 2. State-of-the-art and aim of the study

In the present study, the technology transfer of an earlier zeolite-based sensor concept [7-9] that employed a combination of thin-film processes, photolithography, and thick-film processes to a robust device manufactured without thin-film and lithography processes shall be studied. It is the aim of this study to demonstrate that the technology transfer was successful, to obtain initial data on the long-term stability of the senor devices, and to test its cross sensitivity towards several gas components that might be present in real exhaust.

Zeolites are aluminosilicates built up with SiO_4_ and AlO_4_ tetrahedra building blocks that form a ring structure. They form 3-dimensional (3D) frameworks with linked channel systems and well-defined micro- and mesopores and provide an open porosity that gives rise to an exceptionally high surface area. Aluminum ions replacing silicon ions introduce a negative charge into the framework. This charge needs to be compensated by cations that are bound to the host framework but are mobile along the channels [16]. The aluminum ions act as acidic sites to catalyze chemical reactions. Zeolites can also be modified in a post-synthesis step by incorporating catalytically active metal clusters, e.g. Pt or Fe.

Due to their unique property spectrum, zeolites are of high interest in the field of gas sensing [17]. This relatively young but emerging field is reviewed just recently making clear that due to their adsorptivity, high surface area and porosity, presence of mobile ions, and catalytic activity, zeolites are attractive candidates for numerous applications as chemical sensors [18, 19]. Besides the application as a filter layer to improve selectivity (e.g. [20, 21]), they can be used since their electrical film properties (mostly electrical impedance) change directly and selectively when an analyte is present in the base gas [22]. This was demonstrated e.g. for hydrocarbons [23], for ammonia [24], and for water vapor [25, 26]. Recently, it has even been demonstrated that a zeolite ammonia sensor is suitable for applications in the exhaust [27].

**Figure 1.**
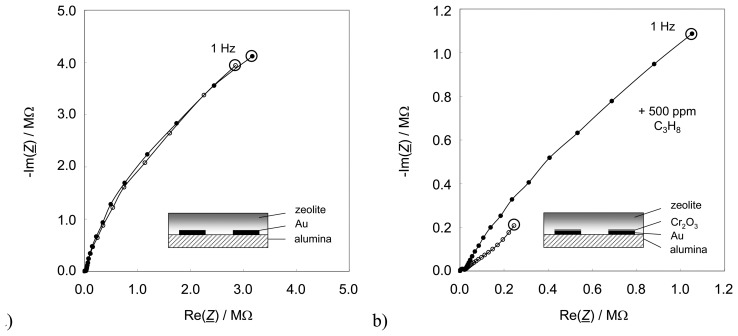
Data of the sensor as it is state-of art (modified from [28]). Nyquist plots of measurements in base gas and with 500 ppm C_3_H_8_ added to the base gas of 2.5 % H_2_O and 10 % O_2_ in N_2_. *T* = 300 °C. **(a)** without Cr_2_O_3_ layer on the IDE **(b)** with Cr_2_O_3_ interfacial layer.

The original impedimetric device as presented in [8, 9] was built up on photolithographically patterned photo resist that covered thin-film alumina substrates. Cr (25 nm) and Au (100 nm) were deposited by thermal evaporation. A subsequent lift-off process lead to interdigital electrodes with a resolution of 20 μm (line = space = 20 μm). After gold sputtering, the interdigital electrode area was covered completely by a screen-printed platinum-loaded zeolite film (Pt-ZSM-5). During the firing process of the zeolite paste, chromium diffused through the gold, got oxidized, and formed a thin Cr_2_O_3_ film [8]. As suggested in [8] and as clearly demonstrated in a subsequent study [28], the Cr_2_O_3_ interfacial layer between Au electrodes and zeolite functional material is essential for the gas-dependent sensor behavior. The influence of the Cr_2_O_3_ film on the zeolite-based impedimetric sensor is clearly demonstrated in Fig. 1, a result of the previous study [28] showing the state-of-the-art. Two sensors are shown, one without and one with Cr_2_O_3_ interfacial layer (Fig. 1a and Fig. 1b, respectively). Due to the preparation in lift-off technique, in the latter case, the spaces between the fingers of the interdigital electrodes are not Cr_2_O_3_-covered. The respective sensor setup is diagrammatically shown in the sketches (insets). The sensors were operated in a tube furnace at 300 °C in a base gas mixture of 2.5 % water vapor and 10 % oxygen. 500 ppm propane were added when indicated.

The sensor effect is a strong increase of the low frequency impedance when hydrocarbons are admixed. It occurs only in the presence of a metal oxide interfacial layer between the zeolite and the electrodes (Fig. 1b). In these initial studies, this very thin layer of Cr_2_O_3_ developed during the standard preparation process of the gold electrodes, where chromium is used as a bonding agent. By XPS analyses it has been proven that a closed chromium oxide film occurred on top of the gold interdigitated electrodes during the firing step of the zeolite thick-film paste [28]. If the zeolite/Cr_2_O_3_ interface is missing (Fig. 1a), the sensor is not sensitive to hydrocarbons at all. As shown in [8], an additionally sputtered Cr_2_O_3_ layer between zeolite and electrode of 100 nm thickness increases the effect. Operated at a constant frequency, e.g. at 1 Hz as indicated by the circles in Fig. 1, such sensor devices show sensitivities up to several hundred percent, if one evaluates the changes in the absolute value of their impedances [8, 9]. Furthermore, it was found out that such sensors are almost insensitive towards hydrogen, carbon monoxide, nitrogen oxide, carbon dioxide, and oxygen (if available in excess) [8, 9].

In order to reduce the manufacturing costs of this sensor, it is aimed to dispense with the costly photolithographic and thin-film processes. Screen-printing the interdigital electrodes would not only make the processes more simple but would additionally lead to thicker gold films which promise an improved long-term stability in rough exhaust atmospheres. Additionally, the use of thick-film substrates lead to a further cost-reduction compared to the thin-film substrates with their low roughness. Hence, the new sensor device is entirely realized in thick-film technology with screen printed structures and electroplated chromium which is subsequently oxidized. An additional screen-printed heating structure on the sensor backside allows to directly control the device temperature. It will be shown that this new configuration provides not only a behavior that is equivalent to the initial devices as shown in Fig. 1, but provides also a good long-term stability.

## 3. Experimental

Gold interdigital electrodes (lines = spaces = 100 μm) were screen-printed with DuPont 5744L paste on alumina substrates (CeramTec Rubalit 708 S) and fired at 850 °C. Electroplated chromium was applied in a chromic acid (H_2_CrO_4_) electrolyte (Atotech CR 843) and subsequently oxidized to Cr_2_O_3_. A current of 30 mA with a maximum voltage of 3 V was applied to the interdigital electrodes for 20 s and the hexavalent Cr^6+^ is deposited as elemental Cr on top of the electrodes. The following oxidation occurred at 600 °C for 10 h in oxygen atmosphere. According to [29], the entire conversion to Cr_2_O_3_ should be completed under these conditions. This was approved by the color change from silver-gray to greenish. The thickness of the Cr coating could not be measured by the available tracing stylus instrument. In SEM fraction pictures, a Cr_2_O_3_ film thicknesses below 100 nm could be estimated. This is less than calculated from the integrated current, even if one takes into account the increase of volume during oxidation the thickness.

**Figure 2.**
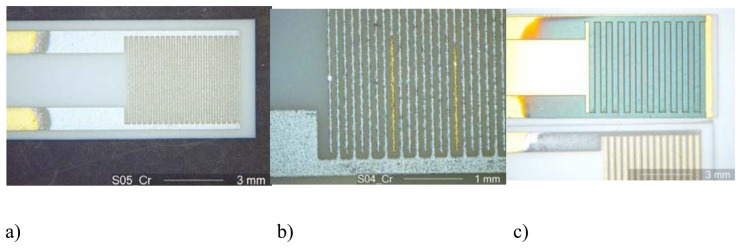
Micrograph of the sensor during the chromium electroplating and oxidation process. **(a)** electroplated interdigital electrode area **(b)** details with an erroneous structure **(c**) with oxidized Cr_2_O_3_ layer.

Figure 2a shows a chromium electroplated interdigital electrode area of a sensor before oxidation. The electroplating process also helps to detect errors in the interdigital electrode structures, as shown in Fig. 2b. Here, a light-optical micrograph of electroplated electrode fingers is shown. One clearly identifies by its color an interruption of a finger, which may be caused by an erroneous screen-printing process. The interrupted fingers are not chromium-covered and remain golden. In Fig. 2c, an oxidized chromium film is shown. The sensor shown on the bottom of the picture represents the pure chromium film, whereas the interdigital electrode area (above) is oxidized. Remarkable is the color change from silver to greenish due to the oxidation to Cr_2_O_3_.

A commercial Na-ZSM-5 zeolite (SüdChemie, SN 27, SiO_2_/Al_2_O_3_ = 27) was platinum-loaded by wet ion-exchange according to [30]. A solution of (Pt(NH_3_)_4_Cl_2_*H_2_O) in water was used and the Na-ZSM-5 powder was added and stirred for 24 hours at room temperature. The dried powder was reduced in a H_2_/N_2_ gas flow in a fluid bed at 450 °C. Highly dispersed platinum clusters inside the zeolite structure were formed. In order to prepare a screen printable paste, organic compounds (Zschimmer & Schwarz KD2721) were added. After screen-printing, the organics were removed by a heat treatment at 450 °C for 6 hours.

Additionally, on some sensor elements a screen-printed heater structure was applied on the backside of the substrate before printing the gold interdigital electrodes. This platinum heater (Heraeus LPA 88/11 S) was covered by a thin ceramic insulation layer to avoid catalytically activated reactions at the hot platinum surface. The entire setup resembled the ammonia sensor in [27]. The temperature homogeneity at 350 °C was determined by an infrared camera and was found out to be better than ± 5 °C over the entire interdigital electrode area.

Measurements of the sensor properties were either conducted in a tube furnace as described in [31] (passively heated operation mode), where the specimens were exposed to different gas compositions. Alternatively, they were operated in the self-heated mode in a gas-flown sensor test bench. The impedance spectra were recorded by a Novocontrol Alpha-Analyzer in the frequency range from 1 Hz to 10 MHz with a voltage amplitude of 40 mV when passively operated and of 500 mV in the self-heated mode. Base gas for the measurements was 20 % O_2_ and 2.5 % H_2_O, all balanced in N_2_.

In the self-heated mode, the sensor was temperature-controlled using the heater resistance as the control variable. The correlation between sensor temperature and heater resistance was generated using a pyrometer. As a result, in the self-heated operation mode, the sensor could be inserted in a test chamber with a gas exchange time of only 5 s. So, it could be ensured that the measured sensor response times are not determined by the sensor test bench.

## 4. Results

**Figure 3.**
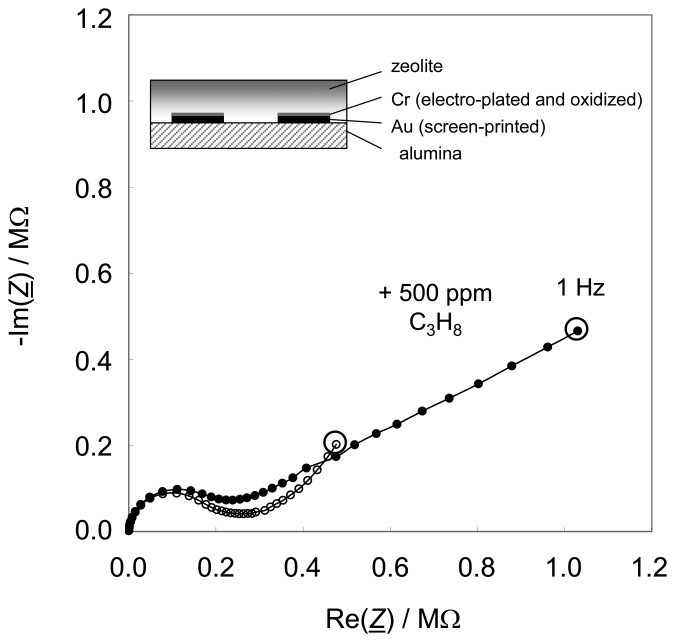
Nyquist plot of a thick film sensor with and without 500 ppm C_3_H_8_ at 350 °C, impedance measured from 1 Hz to 10 MHz, 40 mV amplitude.

Figure 3 shows a typical impedance spectrum of such sensors. The data are taken in a tube furnace at 350 °C in a base gas of 10 % O_2_ and 2.5 % H_2_O in N_2_. All Nyquist plots show a semicircle at high frequencies and a low frequency “tail”. The sensor impedance signal is similar to the one in Fig. 1, which was obtained with a sensor manufactured in the initial technology. In the complex plane, the impedance shows a hydrocarbon-dependent part at low frequencies. At higher frequencies, a semicircle, which is attributed to the volume properties of the zeolite, occurs. Its size and shape does not depend significantly on the hydrocarbon concentration in the base gas. A sensor without electroplated Cr_2_O_3_ film was also prepared and tested. In agreement with literature [32], no hydrocarbon-dependent low-frequency “tail” was observed.

This behavior verifies in principle the sensor functionality. It even demonstrates the successful integration of a Cr_2_O_3_ interfacial layer on the electrode surface by electroplating and subsequent oxidation.

To find out the best operating temperature with respect to high sensitivity and fast sensor response, the impedance response to sudden hydrocarbon changes was evaluated at different temperatures from 250 °C to 400 °C. The measured impedances for each temperature are normalized to compare the signal height and the response times. The sensitivity *S* is defined as the relative change of the absolute value of the impedance at a fixed frequency when exposed to 500 ppm propane normalized to the initial value (before hydrocarbon admixture).

(1) $$S=\frac{\left|\underline{Z}\right|-\left|{\underline{Z}}_{0}\right|}{\left|{\underline{Z}}_{0}\right|}$$

**Figure 4.**
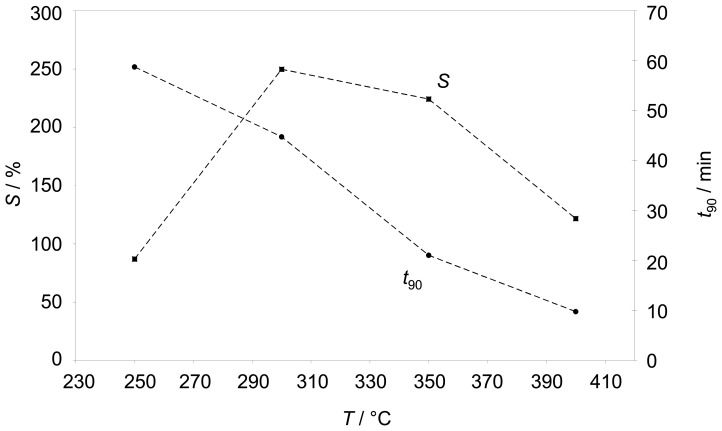
Sensitivity (left axis) and sensor response time (right axis) at a fixed frequency of 3 Hz. Test conditions: base gas 10 % O_2_ and 2.5 % H_2_O in N_2_, switches between 0 ppm and 500 ppm C_3_H_8_ for two hours, temperature varied from 250 °C to 400 °C.

Since the sensor effect appears only at low frequencies, 3 Hz were chosen for the sensitivity calculation. Figure 4 points out how the sensitivity behaves with temperature. In addition, the sensor response time *t*_90_ is shown in Fig. 4. Below 250 °C the sensor is very slow (*t*_90_**≈** 1 h) and the sensitivity *S* is below 100 %. Between 300 °C and 350 °C the sensitivity reaches its maximum, but the impedance still needs ¾ h to reach 90% of its final value. A response time in the range of 10 min occurs at 400 °C, but here the sensitivity gets reduced. As a result, for further tests approx. 350 °C were chosen as a compromise between response time and sensitivity.

**Figure 5.**
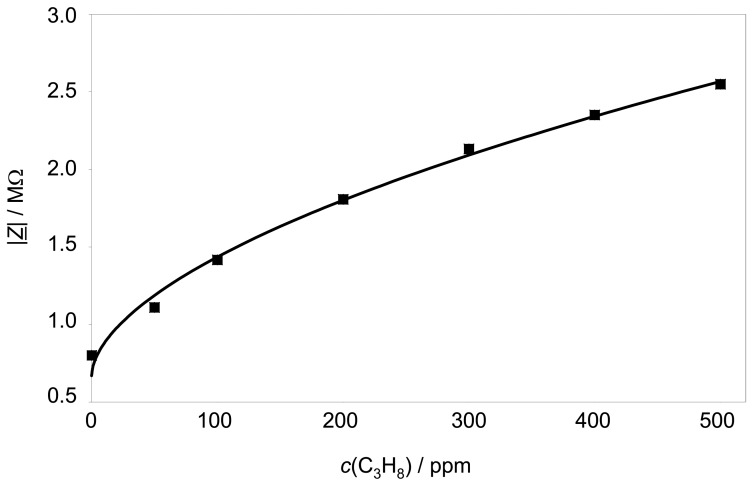
Sensor impedance |*Z̲*| at 3 Hz over the C_3_H_8_ concentration at 350 °C. Base gas 10 % O_2_ and 2.5 % H_2_O in N_2_.

The sensor characteristics (also at 3 Hz) is shown in Fig. 5. The sensor is exposed to different propane concentrations up to 500 ppm propane. |*Z̲*| depends non-linearly with the propane concentration according to equation 2.

(2) $$\left|\underline{Z}\right|/\Omega =a\cdot {\left(c_{\text{HC}}/\text{ppm}\right)}^{b}+z_{0}$$

In equation 2, *c*_HC_ stands for the propane concentration in ppm. The free parameters *a*, *b*, and *z*_0_, can be fitted. In this case the parameters are calculated to:

*a* = 5.7 · 10^4^
*b* = 0.564
*z*_0_ = 6.7 · 10^5^

In other words, the sensor has a high sensitivity at low hydrocarbon concentrations, whereas it gets saturated at higher concentrations.

**Figure 6.**
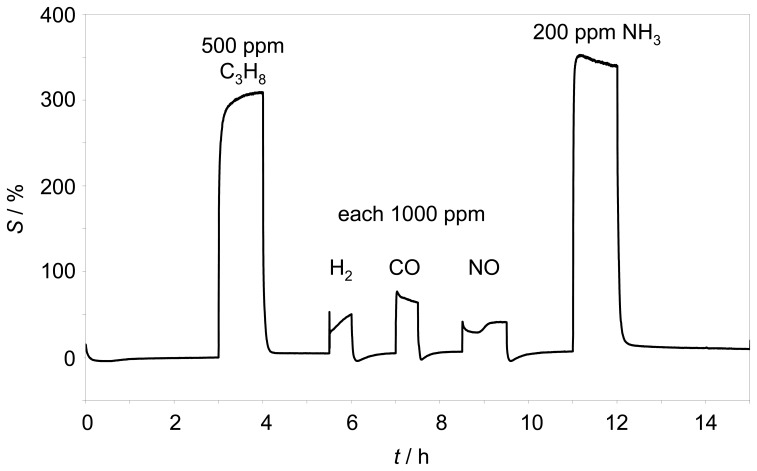
Sensitivity *S* over *t* at 3 Hz and 350 °C.

The selectivity of the initial sensor from [Ref. 8, 9] is high. The magnitude of the complex resistance |*Z̲*| of such a sensor is almost independent towards hydrogen, carbon monoxide, carbon dioxide, and nitrogen monoxide. It has to be evaluated whether with the novel setup a similar low cross-sensitive behavior can be obtained. Therefore, the response behavior towards several test gases was investigated. 500 ppm propane, 1000 ppm hydrogen, 1000 ppm carbon monoxide, 1000 ppm nitrogen monoxide as well as 200 ppm ammonia were added to the base gas as shown in Fig. 6.

The sensor element was operated self-heated at 350 °C. Besides the expected large hydrocarbon effect, only small cross sensitivities to hydrogen, carbon monoxide, and nitrogen monoxide were found. However, the very strong response to ammonia, which had not been tested previously, was astonishing. Compared to the response to propane, the sensor detects ammonia with a higher sensitivity. This can be detrimental for some applications, especially in the exhaust, where ammonia can be formed in reducing atmospheres by the TWC or is added to the exhaust for SCR purposes [4].

**Figure 7.**
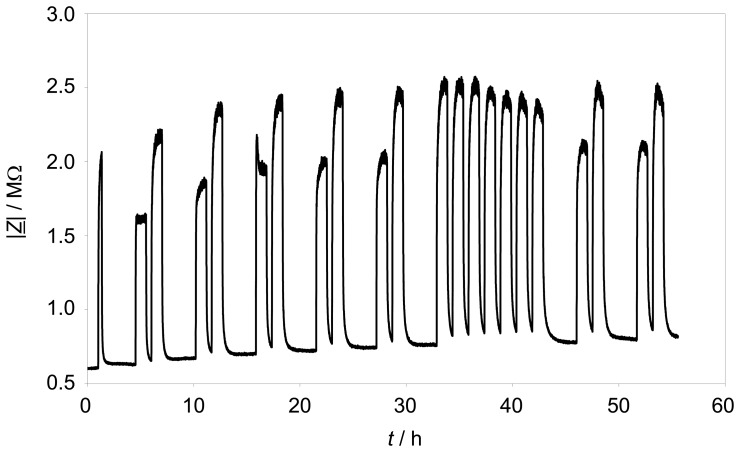
Long-term measurement of |*Z̲*| at 3 Hz and 330 °C. Pulses (duration 1 h, with the exception of the first one (20 min)) of 250 ppm and 500 ppm C_3_H_8_ were applied to the base gas of 10 % O_2_ and 2.5 % H_2_O in N_2_.

Long-term stability is a key issue of a sensor, if one thinks in terms of transforming this concept to series production. Therefore, pulses of 250 ppm and 500 ppm propane were added over almost 2 ½ days to the base gas. During the whole time, the sensor element was self-heated to a constant temperature of 330 °C. Figure 7 shows the trace of |*Z̲*| at the fixed frequency of 3 Hz. The effect of propane is quite constant over 55 hours testing time. A minor rise of the impedance base line is observable, but the hydrocarbon response range is quite constant. The standard deviation of the maximum value of |*Z̲*| with 500 ppm propane is about 2.35 %.

## 5. Discussion

At the moment, the sensor effect is under investigation. Fischerauer et al. set up a theoretical model that takes into account the ionic conductivity of the zeolite, the p-type semiconductor properties of Cr_2_O_3_, and the blocking electrode characteristics of the zeolite/Cr_2_O_3_ interface [34]. The impedance spectrum was calculated using the charge carrier density in the Cr_2_O_3_ film as the key parameter influencing the sensor impedance. It seems that the selectivity is a result of the combination of the zeolite film acting as a filter (as already known from literature [21], [33]) and selective redox reactions at the gas/chromium oxide interface. Hydrocarbons in the ambient modulate the electronic charge carrier density in the Cr_2_O_3_ film due to the same redox-reactions that lead to the simple resistance change effects in p-type semiconducting Cr_2_O_3_ when exposed to reducing gases [35, 36]. Therefore, the sensor impedance responds to the gas concentration changes. Since one knows that resistive chromium oxide-based gas sensors are also cross-sensitive to ammonia (e.g. as known from Cr_2-x_Ti_x_O_3_ (CTO) [37]), it is assumed that the ammonia response of the present sensor is also due to the redox-reactions of Cr_2_O_3_ with reducing gases.

The sensor mechanism is different from recent publications by Mann et al. [38, 39]. These authors used Cr_1.95_Ti_0.05_O_3_ (CTO) as a semiconducting material and applied a chromium or a molybdenum-loaded zeolite filter on top of the semiconducting sensor to discriminate linear alkanes (C7-C11 in [38, 39]). This is in good agreement with recent studies on zeolite filter films on top of hydrocarbon sensitive Sr_1-x_Ti_x_FeO_3_ films [40], where it was shown that a platinum-loaded zeolite filter makes the sensors more sensitive to linear alkanes (C2-C4 in [40, 21]) but reduces the cross-sensitivity to reactive gases like CO and propene. It is interesting to notice that the effect of the zeolite filter is differently explained in the literature. In [21] and [40], redox-reactions in the catalytically active zeolite are attributed to be responsible for the higher selectivity, whereas in [38] the effect of chromium or molybdenum ion exchange is assumed to affect the zeolite channels and dimensions. Since the introduced metal ions vary the pore dimensions, this may lead to more “tortuous paths” for the alkane molecules [38].

One may argue that the exchanged Pt complex replaces Na^+^ and upon thermal decomposition under hydrogen, H^+^ is introduced into the zeolite lattice to maintain charge neutrality. Hence, the zeolite becomes eventually a partial proton conductor [41, 42]. Since these protons become more mobile when ammonia is present in the analyte gas, a possible cross-sensitivity towards ammonia might result, as found out for the zeolite-based ammonia sensor as described in [16, 24, 27]. In this case, a volume effect is the reason for the ammonia sensitivity and one would expect a decreasing high-frequency semicircle as shown for instance in [27]. In addition, the low-frequency impedance should decrease with ammonia admixing. However, an impedance spectrum that looked very similar to the one in Fig. 3 is observed (no figure shown). The high frequency semicircle remains unaffected by ammonia, whereas the low frequency “tail” increases strongly, very similar to the one in Fig. 3. Hence, it seems that the effect leading to the ammonia cross-sensitivity is also based on redox reactions at the gas/chromium oxide interface.

## 6. Conclusions and further work

A novel zeolite-based impedimetric hydrocarbon gas sensor principle, which was originally manufactured in a costly combination of photolithography, thin-film processes, and thick-film processes, was successfully transferred to a low-cost technology comprising only thick-film technology and one electroplating step. The sensor was self-heated to around 350 °C, the optimum operation temperature with respect to sensitivity and response time. Best results are obtained at a low measurement frequency of 3 Hz. The sensor remains stable for at least 55 h. The good selectivity of the original sensor was confirmed, but additionally a very strong cross-sensitivity to ammonia was found, which might prohibit its original intention for use in automotive exhausts. For applications as inexpensive threshold detectors in air, however, these sensors might be of interest, but only if one can exclude ammonia interferences.

## References

[1] Shelef M., McCabe R. (2000). Twenty-five years after introduction of automotive catalysts: what next?. Catalysis Today.

[2] Twigg M.V. (2007). Progress and future challenges in controlling automotive exhaust gas emissions. Applied Catalysis B: Environmental.

[3] Riegel J., Neumann H., Wiedenmann H. M. (2002). Exhaust gas sensors for automotive emission control. Solid State Ionics.

[4] Moos R. (2005). A Brief Overview on Automotive Exhaust Gas Sensors Based on Electroceramics. International Journal of Applied Ceramic Technology.

[5] Sahner K., Fleischer M., Magori E., Meixner H., Deerberg J., Moos R. (2006). HC-sensor for exhaust gases based on semiconducting doped SrTiO_3_ for On-Board Diagnosis. Sensors and Actuators B-Chemical.

[6] Moser T., Stanglmeier F., Schumann B., Thiemann-Handler S. (2000). Sensor in planarer Dickschichttechnik zur Messung von Kohlenwasserstoffen im Abgas von Kraftfahrzeugen (Planar thick-film sensor for measuring hydrocarbons in automotive exhausts).

[7] Plog C., Maunz W., Kurzweil P., Obermeier E., Scheibe C. (1995). Combustion gas sensitivity of zeolite layers on thin-film capacitors. Sensors and Actuators B-Chemical.

[8] Hagen G., Dubbe A., Rettig F., Jerger A., Birkhofer T., Müller R., Plog C., Moos R. (2006). Selective impedance based gas sensors for hydrocarbons using ZSM-5 zeolite films with chromium(III)oxide interface. Sensors and Actuators B-Chemical.

[9] Birkhofer T., Jerger A., Knezevic A., Moos R., Müller R., Plog C., Rettig F., Simon U. (2003). Gassensor und Verfahren zur Detektion von Kohlenwasserstoffen, insbesondere im Abgas von Kraftfahrzeugen. German Patent application.

[10] Higashiyama K., Nagayama T., Nagano M., Nakagawa S. (2003). A catalyzed hydrocarbon trap using metal-impregnated zeolite for SULEV systems. SAE paper 2003-01-0815.

[11] Takahashi A., Noda N., Hiramatsu T., Shibagaki Y. (1998). System for exhaust gas purification. US Patent specification.

[12] Lachman I. (1993). Modified zeolite for trapping hydrocarbons. European Patent application.

[13] T. Minami T. (1989). Exhaust gas purifying apparatus for automobile. US Patent specification.

[14] Endo T., Shimizu H., Motohashi G. (2001). HC-Adsorbent for internal combustion engine. US Patent specification.

[15] Frost J., Bennet S., Lafyatis D., Walker A. (1995). Emission control system for an internal combustion engine. European Patent specification.

[16] Franke M. E., Simon U., Moos R., Knezevic A., Müller R., Plog C. (2003). Development and Working Principle of an Ammonia Gas Sensor based on a Refined Model for Solvate Supported Proton Transport in Zeolites. Phys. Chem. Chem. Phys..

[17] Sahner K., Hagen G., Schönauer D., Reiß S., Moos R. Zeolites - versatile materials for gas sensors. Solid State Ionics.

[18] Xu X., Wang J., Long Y. (2006). Zeolite-based materials for gas sensors. Sensors.

[19] Moos R., Sahner K., Hagen G., Dubbe A. (2006). Zeolites for Sensors for Reducing Gases. Rare Metal Materials And Engineering.

[20] Vilaseca M., Coronas J., Cirera A., Cornet A., Morante J., Santamaría J. (2003). Use of zeolite films to improve the selectivity of reactive gas sensors. Catalysis Today.

[21] Sahner K., Moos R. (2008). Zeolite cover layer for selectivity enhancement of p-type semiconducting hydrocarbon sensors. Sensors and Actuators B-Chemical.

[22] Simon U., Franke M. (2000). Electrical properties of nanoscaled host/guest compounds. Microporous and Mesoporous Materials.

[23] Alberti K., Fetting F. (1994). Zeolites as sensitive materials for dielectric gas sensors. Sensors and Actuators B-Chemical.

[24] Simon U., Flesch U., Maunz W., Müller R., Plog C. (1998). The effect of NH_3_ on the ionic conductivity of dehydrated zeolites Na beta and H beta. Microporous and Mesoporous Materials.

[25] Schäf O., Ghobarkar H., Guth U. (1997). Sensors for combustible gas components using modified single crystal zeolites. Ionics.

[26] Neumeier S., Echterhof T., Bölling R., Pfeifer H., Simon U. (2008). Zeolite based trace humidity sensor for high temperature applications in hydrogen atmosphere. Sensors and Actuators B-Chemical.

[27] Moos R., Müller R., Plog C., Knezevic A., Leye H., Irion E., Braun T., Marquardt K.-J., Binder K. (2002). Selective Ammonia Exhaust Gas Sensor for Automotive Applications. Sensors and Actuators B-Chemical.

[28] Hagen G., Schulz A., Knörr M., Moos R. (2007). Four-wire impedance spectroscopy on planar zeolite/chromium oxide based hydrocarbon gas sensors. Sensors.

[29] Lerm A, Elbinger G., Rosemann P. (1978). Untersuchungen zum pT-Diagramm und zur Stabilität von Chrom(IV)-oxid. Zeitschrift für anorganische und allgemeine Chemie.

[30] Plog C., Maunz W. (1999). Use of a gas sensor for the selective detection of hydrocarbons in low-oxygen gases. US Patent Specification.

[31] Sahner K., Moos R., Matam M., Tunney J.J., Post M. (2005). Hydrocarbon sensing with thick and thin film p-type conducting perovskite materials. Sensors and Actuators B-Chemical.

[32] Hagen G., Dubbe A., Fischerauer G., Moos R. (2006). Thick-film impedance based hydrocarbon detection based on chromium(III) oxide / zeolite interfaces. Sensors and Actuators B-Chemical.

[33] Sahner K., Schönauer D., Moos R., Matam M., Post M.L. (2006). Effect of electrodes and zeolite cover layer on hydrocarbon sensing with p-type perovskite SrTi_0.8_Fe_0.2_O_3-δ_ thick and thin films. Journal of Materials Science.

[34] Fischerauer A., Gollwitzer A., Thalmayr F., Hagen G., Moos R., Fischerauer G., Gerlach G., Hauptmann P. Modellierung des Impedanzspektrums eines Kohlenwasserstoffsensors mit einer Zeolith-Cr_2_O_3_-Grenzfläche.

[35] Moseley P.T., Williams D.E. (1989). Gas Sensors Based on Oxides of Early Transition Metals. Polyhedron.

[36] Niemeyer D., Williams D.E., Smith P., Pratt K.F.E., Slater B., Catlow C.R.A., Marshall Stoneham A. (2002). Experimental and computational study of the gas-sensor behaviour and surface chemistry of the solid-solution Cr_2-x_Ti_x_O_3_ (x ≤ 0.5). J. Mater. Chem..

[37] Tofield B.C., Moseley P.T., Norris J.O.W., Williams D.E. (1988). Metal Oxide Gas Sensors. UK patent specification.

[38] Mann D.P., Pratt K.F.E., Paraskeva T., Parkin I.P., Williams D.E. (2007). Transition metal exchanged zeolite layers for selectivity enhancement of metal-oxide semiconductor gas sensors. IEEE Sensors Journal.

[39] Mann D.P., Paraskeva T., Pratt K.F.E., Parkin I.P., Williams D.E. (2005). Metal oxide semiconductor gas sensors utilizing a Cr-zeolite catalytic layer for improved selectivity. Measurement Science and Technology.

[40] Sahner K., Schönauer D., Matam M., Post M., Moos R. (2008). Selectivity enhancement of p-type semiconducting hydrocarbon sensors - the use of sol precipitated nano-powders. Sensors and Actuators B-Chemical.

[41] Kanellopoulos J., Gottert C., Schneider D., Knorr B., Prager D., Ernst H., Freude D. (2008). NMR investigation of proton mobility in zeolites. Journal of Catalysis.

[42] Franke M.E., Sierka M., Simon U., Sauer J. (2002). Translational proton motion in zeolite H-ZSM-5. Energy barriers and jump rates from DFT calculations. Phys. Chem. Chem. Phys..

